# Ways to unravel the clinical potential of carbon ions for head and neck cancer reirradiation: dosimetric comparison and local failure pattern analysis as part of the prospective randomized CARE trial

**DOI:** 10.1186/s13014-022-02093-4

**Published:** 2022-07-08

**Authors:** Thomas Held, Thomas Tessonnier, Henrik Franke, Sebastian Regnery, Lukas Bauer, Katharina Weusthof, Semi Harrabi, Klaus Herfarth, Andrea Mairani, Jürgen Debus, Sebastian Adeberg

**Affiliations:** 1 Department of Radiation Oncology, Heidelberg University Hospital, Im Neuenheimer Feld 400, 69120 Heidelberg, Germany; 2 Heidelberg Institute of Radiation Oncology (HIRO), Heidelberg, Germany; 3 National Center for Tumor Diseases (NCT), Heidelberg, Germany; 4 Clinical Cooperation Unit Radiation Oncology, German Cancer Research Center (DKFZ), Heidelberg, Germany; 5 Heidelberg Ion Beam Therapy Center (HIT), Heidelberg, Germany; 6 German Cancer Consortium (DKTK), partner site Heidelberg, German Cancer Research Center (DKFZ), Heidelberg, Germany; 7 Medical Physics, National Centre of Oncological Hadrontherapy (CNAO), Pavia, Italy

**Keywords:** Heavy ions, Carbon ion radiotherapy, Squamous cell carcinoma, Reirradiation, Head and neck cancer, Local control, Dosimetric analysis, Pattern of failure, Re-radiotherapy, VMAT

## Abstract

**Background:**

Carbon ion radiotherapy (CIRT) yields biophysical advantages compared to photons but randomized studies for the reirradiation setting are pending. The aim of the current project was to evaluate potential clinical benefits and drawbacks of CIRT compared to volumetric modulated arc therapy (VMAT) in recurrent head and neck cancer.

**Methods:**

Dose-volume parameters and local failure patterns of CIRT compared to VMAT were evaluate in 16 patients from the randomized CARE trial on head and neck cancer reirradiation.

**Results:**

Despite an increased target dose, CIRT resulted in significantly reduced organ at risk (OAR) dose across all patients (− 8.7% Dmean). The dose-volume benefits were most pronounced in the brainstem (− 20.7% Dmax) and the optic chiasma (− 13.0% Dmax). The most frequent local failure was type E (extraneous; 50%), followed type B (peripheral; 33%) and type A (central; 17%). In one patient with type A biological and/or dosimetric failure after CIRT, mMKM dose recalculation revealed reduced target coverage.

**Conclusions:**

CIRT resulted in highly improved critical OAR sparing compared to VMAT across all head and neck cancer reirradiation scenarios despite an increased prescription dose. Local failure pattern analysis revealed further potential CIRT specific clinical benefits and potential pitfalls with regard to image-guidance and biological dose-optimization.

**Supplementary Information:**

The online version contains supplementary material available at 10.1186/s13014-022-02093-4.

## Background

Carbon ion radiotherapy (CIRT) yields well-known biophysical advantages [1] compared to photons yet what are key clinical benefits and potential pitfalls for patients with recurrent head and neck cancer? Although recent studies reported excellent outcomes with CIRT in various recurrent tumor sites [2], high-level evidence of clinical superiority is pending. Besides careful patient selection, the complex reirradiation setting requires a high-precision approach to enhance the balance of tumor control and toxicity. Therefore, the randomized controlled CARE trial [3], comparing CIRT with volumetric modulated arc therapy (VMAT) for reirradiation of head and neck cancer, was recently initiated.

Building on previous photon reirradiation studies [4, 5], modern conformal radiation techniques such as stereotactic body radiation therapy improved outcomes primarily in early-stage recurrent tumors [6]. In reality, most inoperable recurrences are locally advanced and target volume delineation is further impeded by tissue changes from previous treatments, making VMAT the standard of care [7]. Both proton [8] and in particular carbon ion reirradiation [9, 10] could further enhance dose escalation while reducing normal tissue complication probabilities. However, sophisticated radiobiological models [11] are required to calculate the relative biological effectiveness (RBE)-weighted dose of carbon ions for different tissues, treatment parameters and endpoints. Biological dose calculation based on the local effect model [12, 13] (LEM) I depends primarily on the clinical α/β value from the linear quadratic model, the absorbed dose and the underlying complex mixed radiation field of carbon ions to predict the local RBE. More recent RBE-models, in particular the modified microdosimetric kinetic model [14] (mMKM), revealed trends towards superior RBE prediction in low/midrange LET conditions [15] but outcome analysis in clinical trials is pending. Moreover, treatment delivery uncertainties of scanned carbon beams, mitigated by image-guidance and robust optimization, are crucial for treatment planning.

In the current project, dose-volume parameters of CIRT and VMAT were compared for target volumes and organs at risk (OAR) in different reirradiation scenarios. Further implications for target volume delineation and dose optimization were evaluated as part of a pattern of local failure analysis. Thereby, the aim of the current project was to unravel potential clinical benefits and pitfalls of CIRT compared to VMAT in patients with recurrent head and neck cancer.

## Methods

### Patient selection

After approval by the regional ethics committee (S-708/2018), the prospective randomized CARE trial was initiated in 2020. In this study [3], patients with recurrent head and neck cancer receive reirradiation with either CIRT or VMAT. We selected the first 16 patients enrolled in the study to compare dose-volume parameters and evaluate local failure patterns.

### Reirradiation treatment planning

Treatment planning was conducted according to the CARE protocol [3] using RayStation 10A (RaySearch Laboratories, Stockholm, Sweden). Delineation of OAR was performed in line with consensus guidelines [16]. The gross tumor volume (GTV) was defined based on contrast-enhanced computed tomography (CT), magnetic resonance imaging (MRI) and surgery/pathology reports. The clinical target volume (CTV) included the macroscopic tumor and/or tumor bed with a margin of 3–5 mm. A margin of 2–3 mm was added for the planning target volume (PTV). No elective nodal irradiation was performed.

All patients treated with VMAT received 60 Gy in 30 fractions (5 fractions/week). Patients that received CIRT were treated with 51 Gy (RBE) in 17 fractions (5–6 fractions/week) or 51–60 Gy (RBE) if the radiotherapy (RT) interval was ≥ 2 years. Additional 20–30% total cumulative dose to OAR was only allowed after a minimum of two years [17, 18]. Calculation of the RBE-weighted dose of CIRT was based on LEM I [12, 19] with an α/β value of 2 Gy for the target and OAR, in line with the clinical standard at our institution.

Image-guidance consisted of daily orthogonal X-rays for CIRT [20] with additional CT scans in selected patients or daily kV cone-beam CTs for VMAT. If required, the treatment plan was adapted at the discretion of the radiation oncologist team. Treatment delivery for CIRT was performed with a raster scanning delivery at a synchrotron-based facility using active energy selection [21], while using a 6 MV linear accelerator for VMAT treatment.

### Dosimetric analysis and comparison

For all patients that received VMAT, an additional CIRT treatment planning in line with the CARE protocol was performed and vice versa. Dose-volume parameters were extracted for the target volumes and most relevant OAR. Target volume coverage was assessed based on the volume (in %) receiving ≥ 95% of the prescribed dose (V95%). The dose distribution was evaluated using the homogeneity index [22] for the CTV, where the ideal value is zero. The maximum (Dmax) and mean (Dmean) dose and for all OAR in addition the dose applied to 1% (D1), 5% (D5) and 10% (D10) was specified. All CIRT treatment plans were scaled down to 51 Gy (RBE) total dose to facilitate comparison. The applied dose was specified by the equivalent dose in 2 Gy fractions (EQD2) for all patients. All dose reductions were specified further in percent of 60 Gy EQD2 for both CIRT and VMAT to facilitate comparisons. Predefined clinical goals for reirradiation, depending on the previous RT, were evaluated for target volumes and OAR according to the study protocol. Mean dose volume histograms were generated for all regions of interest.

### Pattern of local failure evaluation

In patients with local failure after reirradiation, the recurrent macroscopic tumor was contoured on the diagnostic CT/MRI, mapped to the planning CT and deformed manually (rGTV). In three patients, more than one rGTV was delineated. Based on the rGTV, the recurrence volume, Dmean, Dmax and D95% were evaluated. Modified from Mohamed et al. [23], local failure was evaluated by centroid (central voxel of rGTV with 2 mm margin) location in relation to the CTV. Failures were classified in type A (central CTV; D95% rGTV ≥ 95% prescribed dose), type B (peripheral CTV; D95% rGTV < 95% prescribed dose) and type E (outside CTV). In patients with type A local failure after CIRT, dose recalculation based on mMKM was conducted.

### Statistical methods

Statistical analysis was performed using R version 4.1.0 (www.r-project.org). For each dose-volume parameter, the mean and standard deviation was compared. The Wilcoxon signed-rank test was applied to evaluate differences based on paired samples (CIRT vs. VMAT). A *p* value < 0.05 was considered statistically significant.

## Results

### Patient and treatment characteristics

All patients had locally advanced recurrent tumors, most of them high-grade (63%). The median age prior to reirradiation was 59 years (range 49–73 years) and the median RT interval was 3.3 years (range 0.7–28.0 years). The majority were male patients (69%) and/or active smokers (63%). Most patients had squamous cell carcinoma (81%) and received definitive RT (75%). The mean GTV, CTV and PTV were 28.9, 69.5 and 107.5 ccm and the mean cumulative EQD2 was 130.1 Gy. A total of nine patients received CIRT and seven VMAT. Relevant patient characteristics are summarized in Table 1.

**Table 1:** Patient characteristics, tumor classification and treatment of all patients with recurrent head and neck cancer

Pat	ReRT	Age (y)	KPS (%)	Localization	Type	Concept	T	N	M	R	G	Interval (y)	ReRT EQD2 (Gy)	Cum. EQD2 (Gy)	CTV (ccm)
01	CIRT	54	70	Oral cavity	SCC	Postop	4	0	0	2	2	1.0	75.0	129.0	167.9
02	CIRT	57	90	Nasopharynx	SCC	Def	2	0	0		2	0.7	63.8	137.8	30.6
03	CIRT	61	90	Hypopharynx	SCC	Def	4	0	0		3	3.6	63.8	123.8	84.0
04	VMAT	72	80	Sinuses	SCC	Def	4	0	0		2	1.0	60.0	126.0	110.0
05	CIRT	73	70	Oral cavity	MEC	Def	4	0	0		3	1.0	67.5	147.5	25.0
06	VMAT	71	80	Oropharynx	SCC	Def	4	0	0		2	7.2	60.0	123.0	65.0
07	VMAT	54	80	Nasopharynx	SCC	Def	1	2	0		3	4.5	60.0	124.0	54.7
08	CIRT	54	90	Sinuses	AC	Postop	3	0	0	2	3	1.6	67.5	127.5	49.3
09	VMAT	52	80	Skull base	AC	def	4	0	0		3	0.9	60.0	130.0	10.4
10	VMAT	49	80	Nasopharynx	SCC	def	4	0	0		3	3.3	60.0	124.0	31.7
11	VMAT	65	80	Nasal cavity	SCC	Def	3	0	0		3	4.0	60.0	130.0	37.4
12	VMAT	55	80	Nasal cavity	SCC	Postop	3	0	1	1	2	1.7	60.0	120.0	25.6
13	CIRT	62	70	Oral cavity	SCC	Def	3	0	0		2	8.3	63.8	137.8	42.4
14	CIRT	69	70	Oral cavity	SCC	Def	4	0	0		3	28.0	63.8	138.8	196.4
15	CIRT	50	90	Nasal cavity	SCC	Postop	4	0	0	2	3	1.2	63.8	129.8	139.6
16	CIRT	69	80	Oropharynx	SCC	Def	4	0	0		3	17.9	67.5	133.5	42.6

*CIRT* carbon ion radiotherapy, *VMAT* volumetric modulated arc therapy, *KPS* Karnofsky Performance Score, *EQD2* equivalent dose in 2 Gy fractions, *ReRT* re-radiotherapy, *GTV* gross tumor volume, *CTV* clinical target volume, *PTV* planning target volume, *f* female, *m* male, *SCC* squamous cell carcinoma, *MEC* mucoepidermoid carcinoma, *AC* adenocarcinoma

### Dosimetric analysis of target volumes and organs at risk

**Fig. 1:**
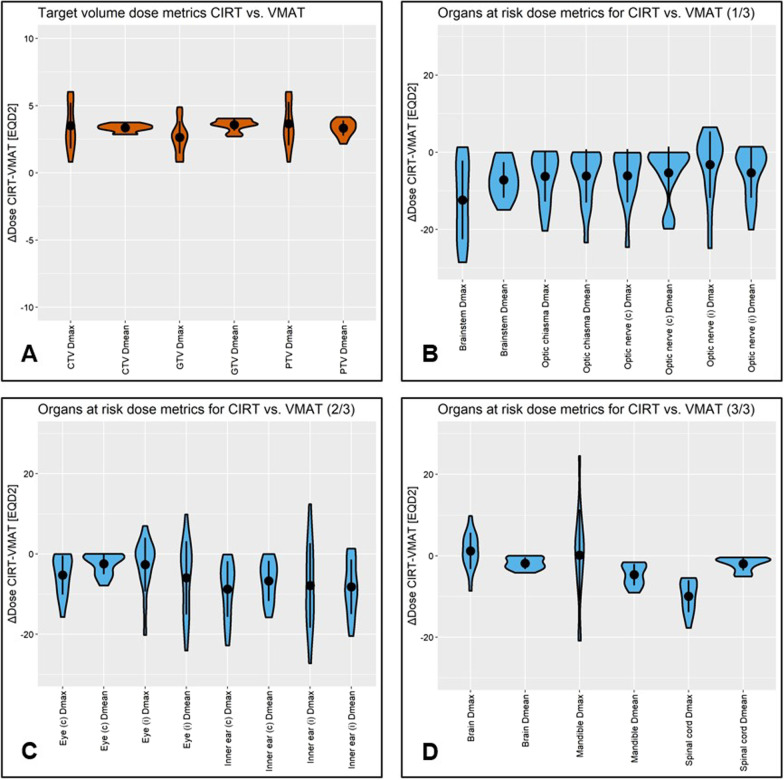
Dose-volume metrics are specified as ΔCIRT-VMAT in equivalent dose in 2 Gy fractions (EQD2) for the target volumes and all relevant organs at risk. The mean and standard deviation are shown for each dose parameter within the violin plot. Despite the increased target dose (+ 5.6% CTV Dmean; *p* < 0.001), CIRT resulted in significantly reduced organ at risk dose across all patients (− 8.7% Dmean) compared to VMAT

The mean EQD2 in the CTV was 63.6 Gy for CIRT and 60.2 Gy for VMAT (*p* < 0.001). Target volume coverage was comparable with both modalities. The V95% of the GTV, CTV and PTV was not significantly different between CIRT and VMAT. The homogeneity index of the CTV was reduced with CIRT compared to VMAT (*p* = 0.003). Target volume and OAR dose metrics of CIRT vs. VMAT are shown in Fig. 1A. Further data is shown in the appendix (Additional file 1: Tab. S3).

**Fig. 2:**
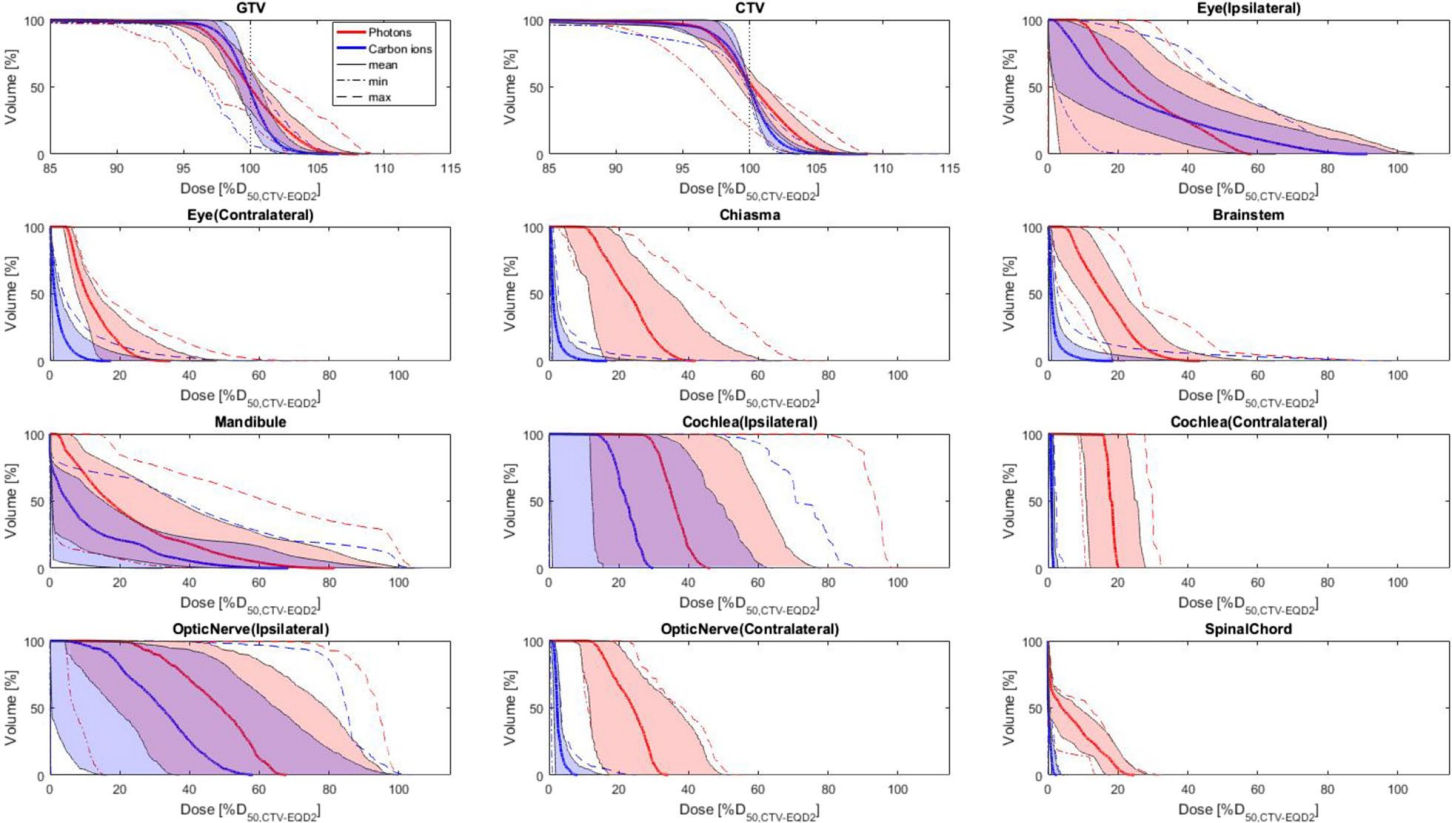
Cumulative mean dose–dose-volume histograms (DVH) reveal superiority for the target and all organs at risk for CIRT compared to VMAT. The dose is specified as percent of the prescribed equivalent dose in 2 Gy (EQD2) fractions to facilitate comparison. Mean dose–DVH as well as standard deviation, minimum/maximum DVH for several regions of interest are presented

**Fig. 3:**
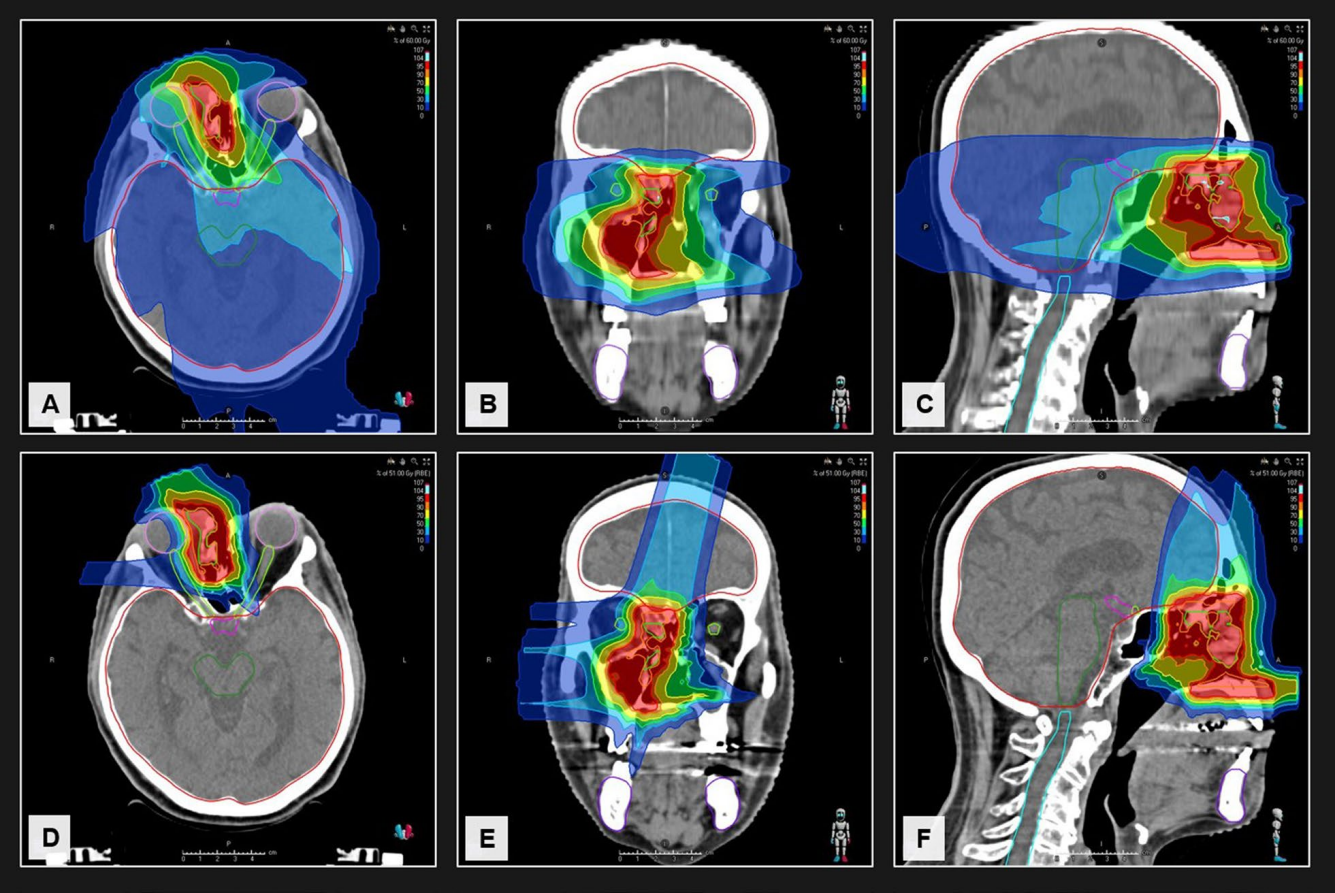
65-year-old male patient with nasal cavity recurrence treated with 60 Gy VMAT (**A**–**C**) around 4 years after previous radiotherapy with 70 Gy. CIRT (**D**–**F**) yielded significant clinical benefits w.r.t. the brainstem (− 31.5% Dmax), the optic chiasma (− 34.0% Dmax), the ipsilateral eye (− 30.5% Dmean) and the contralateral optic nerve (− 28.8% Dmean). The maximum dose in the ipsilateral optic nerve was not reduced but the mean dose (− 16.2%). The patient developed no local failure during follow-up

Despite the significantly higher prescribed dose in the CTV, the mean Dmean throughout all evaluated OAR was significantly reduced with CIRT compared to VMAT (Fig. 1B–D). A pronounced decrease of mean dose occurred among others in the ipsilateral inner ear (− 8.2 Gy EQD2; 13.7%), the brainstem (− 7.2 Gy EQD2; 12.0%) and the optic chiasma (− 6.1 Gy EQD2; 10.2%). Also, the mean dose to the optic nerves and ipsilateral eye was reduced by 8.8 and 9.8%, respectively. Cumulative mean dose-volume histograms for all regions of interest are shown in Fig. 2. Further data is shown in the appendix (Additional file 1: Tab. S4). A clinical case example of a patient with nasal cavity recurrence is shown in Fig. 3.

Several patients had tumors in close proximity or with direct infiltration of these OARs. In a total of three patients, adherence to ipsilateral optic nerve dose constraints was not possible. All three patients decided to accept increased toxicity risks for maximum tumor control probability. Adherence to clinical constraints was comparable with both RT modalities, as summarized in the appendix (Additional file 1: Tab. S5).

### Pattern of local failure analysis

Local failure occurred in 9/14 evaluable patients (64%) within six months after reirradiation. Two of these patients had parallel nodal or distant failure. The most frequent salvage treatment was immunotherapy (67%), followed by salvage-surgery and best supportive care. The mean rGTV was 8.1 ccm (range 1.6–17.5 ccm). The intricacy of local failure evaluation is summarized in Table 2 as a basis for all clinical scenarios available in the appendix (Additional file 1: Fig. 5–13).

**Table 2:** Local failure pattern analysis after reirradiation with CIRT vs. VMAT in recurrent head and neck cancer

Pat	ReRT	Failure type	rGTV (ccm)	rGTV Dmean	rGTV D95%	rGTVc Dmean	N failure	M failure	Possible cause of local failure	Salvage treatment
01	CIRT	E	15.9	30.4	5.7	19.4	No	No	Aberrant areas of recurrence	Best supportive care
02	CIRT	E	4.4	6.2	1.3	2.8	No	No	Aberrant areas of recurrence	Immunotherapy
03	CIRT	A	1.6	63.8	62.8	63.6	No	No	Biological/dosimetric failure	Salvage surgery
04	VMAT	B and E	4.6	59.1/35.3	53.3/10.7	60.4/36.2	No	No	Overgrown recurrence + aberrant areas of recurrence	Immunotherapy
08	CIRT	E	5.4	42.1	6.9	57.9	Yes	Yes	Improper risk assessment	Salvage surgery
09	VMAT	E	6.6	16.5	6.2	13.4	No	No	Aberrant areas of recurrence	Chemotherapy
10	VMAT	B	2.2	37.4	15.0	38.9	Yes	No	Dosimetric failure	Chemotherapy
14	CIRT	A and B	14.8	63.8/59.5	62.8/45.9	63.5/63.0	No	No	Biological/dosimetric failure + overgrown recurrence	Immunotherapy
15	CIRT	B and E	17.5	59.8/0	36.3/0	63.1/0	No	No	Overgrown recurrence + aberrant areas of recurrence	Immunotherapy

CIRT Carbon ion radiotherapy, VMAT volumetric modulated arc therapy, RT Radiotherapy, rGTV Recurrence gross tumor volume, Dmean Mean dose; D95% Dose reached in 95% of the rGTV, rGTVc rGTV centroid, N nodal, M metastatic

**Fig. 4:**
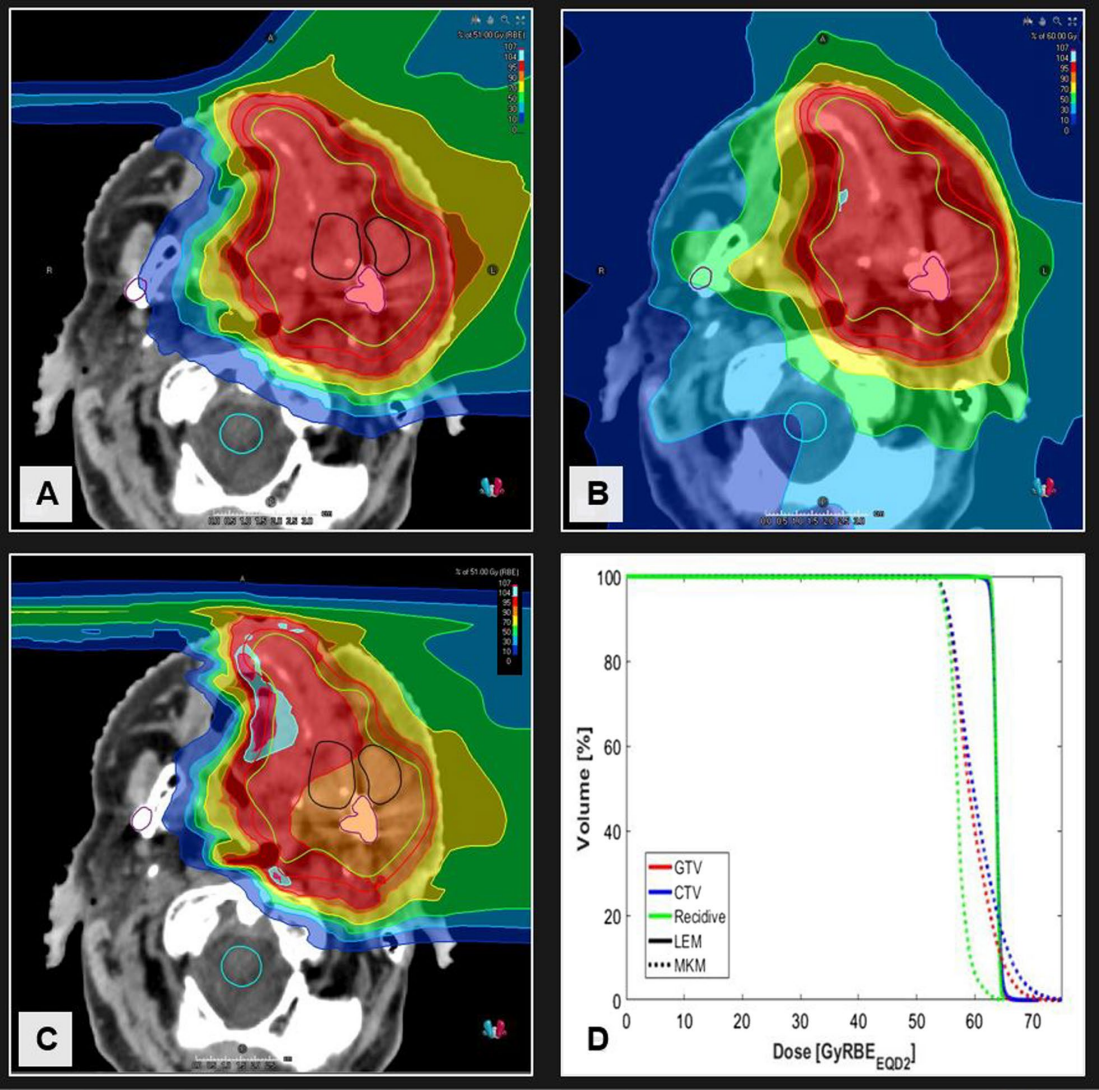
69-year-old female patient with recurrent oral cavity cancer treated with 51 Gy (RBE) CIRT (**A**) around 28 years after prior radiotherapy with 75 Gy. CIRT yielded significant clinical benefits w.r.t. the brainstem (− 47.5% Dmax), ipsilateral optic nerve (− 16.8% Dmax), ipsilateral eye (− 21.3% Dmean) and contralateral inner ear (− 23.2% Dmean) compared to VMAT (**B**). The patient developed type A local failure, delineated on the planning CT (**A**), caused biological and/or dosimetric failure. Dose recalculation with the modified microdosimetric model w.r.t. the type A failure revealed significantly reduced rGTV Dmean and rGTV D95% dose EQD2 compared to the local effect model I (**C**–**D**)

In all nine patients with local failure, in total twelve rGTVs were contoured since three patients had more than one type of local failure simultaneously. The most frequent local failure was type E (n = 6; 50%), followed by type B (n = 4; 33%) and type A (n = 2; 17%). Most rGTV centroids of type E local failures were localized outside CTV + 5 mm (n = 5; 83%). Possible causes of type E local failure were aberrant areas of recurrence (n = 5; 83%) or improper risk assessment (n = 1; 17%). Patients with type B local failure had either overgrown recurrence (n = 3; 75) or dosimetric failure (n = 1; 25%). Potential causes of type A local failure were biological and/or dosimetric failure. Dose recalculation with mMKM in both patients with type A local failure after CIRT revealed significantly reduced rGTV Dmean and rGTV D95% in one patient. A clinical case example of mMKM dose recalculation in a patient with type A failure after CIRT is shown in Fig. 4. Further clinical case examples of all other patients are shown in the appendix.

## Discussion

The biophysical properties of CIRT translated into clinical dose-volume advantages both in the target volume and OAR across all reirradiation scenarios compared to VMAT. Local failure pattern analysis revealed further CIRT specific clinical benefits and potential pitfalls in patients with recurrent head and neck cancer.

Adding on to the complex reirradiation setting, the patient cohort included primarily unfavorable high-grade tumors (63%) with a mean GTV of 29 ccm. The target prescription dose was superior with CIRT compared to VMAT (CTV Dmean: 63.6 vs. 60.2 Gy EQD2; + 5.6%; *p* < 0.001). In general, reirradiation tumor control is dose-dependent and significantly inferior with a prescribed dose ≤ 60 Gy [24]. However, reirradiation with ≥ 68 Gy is associated with excessive toxicity, thereby resulting in decreased survival rates compared to 60 Gy [25]. In our study, most patients (n = 12; 75%) were treated within the recommended range of 60–66 Gy [7] prescription dose and some above (n = 4; 100% CIRT; Dmean 69.4 Gy EQD2). CIRT may enable further dose escalation without increasing side effects due to its biophysical advantages compared to VMAT, but outcome evaluation is pending. In comparison to squamous cell carcinoma (SCC), dose escalation effects may have more pronounced impact on tumor control in radioresistant tumors such as adenoid cystic carcinomas [26, 27]. Moreover, the previously described risk of severe soft-tissue necrosis [28] in the CIRT high-dose area of the previously irradiated target remains problematic. In recurrent SCC, the impact of moderate hypofractionated CIRT compared to normofractionated VMAT on tumor control and normal tissue complication probability (NTCP) has yet to be determined in randomized clinical trials.

Despite the increased target dose, CIRT resulted in significantly reduced OAR dose across all patients (− 8.7% Dmean) compared to VMAT. Reirradiation NTCPs have yet to be determined in clinical trials, considering previous sequelae, the radiation interval and cumulative dose-volume metrics. The toxicity profiles as the primary endpoint of the CARE trial will therefore be analyzed after completion of the study. Previous dosimetric comparisons of proton RT and VMAT have shown clinical benefits with similar mean dose reductions [29, 30] and NTCPs could be even more dose-dependent in the reirradiation setting. On the other hand, for OAR close to the target, EQD2 reduction by CIRT is attenuated due to hypofractionation compared to normofractionated VMAT.

The dose-volume benefits were most pronounced in the brainstem (− 20.7% Dmax) and the optic chiasma (− 13.0% Dmax) with CIRT compared to VMAT. The mean dose to the ipsilateral and contralateral inner ear (− 11.3%/− 13.7%), eye (− 9.8%/− 4.2%) and optic nerve (− 8.8%/− 9.0%) was also advantageous with CIRT. These dose-limiting OAR are decisive for the treatment option of reirradiation but with exception of the brainstem and spinal cord, a risk–benefit-tradeoff is frequently inevitable [17]. In the current study, the clinical goal of the ipsilateral optic nerve (81.3% vs. 75.0%) was reached slightly more often with CIRT. In line with previous studies on particle therapy [31], the dose reduction was particularly noticeable for the contralateral side with regard to paired OAR (Additional file 1: Tab. S3). The difference in maximum dose did not reach significance for the ipsilateral optic nerve, mandible, brain and ipsilateral eye, due to proximity to the target volume. Other dose-volume metrics (e.g. D1cc) could be more relevant for CIRT in certain clinical scenarios where the high-LET target region is very close to vital OAR.

The pattern of local failure analysis revealed primarily extraneous dose failures (50%), mostly caused by possible aberrant areas of recurrence. Tissue changes from previous local therapies strongly impede target volume delineation in recurrent head and neck cancer. Combining multi-modality imaging including CT, diffusion-weighted MRI and positron emission tomography (PET)/CT can be crucial to mitigate uncertainties in contouring [32, 33]. One patient with type E local failure developed tumor recurrence within 5 mm of the CTV. Due to concerns regarding toxicity, the CTV margin was kept at 3 mm instead of 5 mm, as recommended in the consensus guidelines [7], thereby possibly causing local failure. In patients with type B local failure, the rate of overgrown recurrence could be reduced by improved image-guidance [34], in particular for CIRT. Since the start, ion beam image-guidance was based on daily orthogonal X-rays and weekly CT scans only in selected patients, possibly detecting aggressive tumor growth too late. Current and future perspectives in image-guided adaptive particle therapy, focused on CT/MR imaging [35], can eliminate type B failures caused by overgrown recurrence and provide functional response assessment. In addition, robustness of CIRT can be improved by state-of-the-art image-guidance, mitigating range uncertainties caused by anatomical changes. One type B local failure after VMAT was caused by dosimetric failure, due to proximity to the brainstem. The CIRT treatment plan was non-superior in this scenario, due to the direct spatial relationship of target volume and OAR.

Type A local failure occurred twice after CIRT, potentially caused by dosimetric and/or biological failure in both patients. In one patient the rGTV was located adjacent to a lower jaw metal implant, which potentially caused range uncertainties. In the other patient with hypopharyngeal recurrence, organ motion with changing air/tissue interface possibly deteriorated the dose distribution in the target volume. Moreover, α/β value of 2 Gy may lead to relative underdosage in the target, in particular for patients with SCC (α/β ~ 10 Gy) [36]. Nonetheless, mMKM dose recalculation revealed significantly reduced Dmean and D95% in the rGTV, compared to LEM I, in one patient. These findings advocate further comparisons of RBE-models for CIRT to mitigate RBE uncertainties in the target volume and OAR within the ongoing CARE study. The clinical potential of CIRT is not reached yet. Several further research endeavors, e.g. multi-ion RT [37], hadron arc RT [38], and ultra-high dose rate CIRT [39], aim to further optimize biological effects for the best of the patient.

The current study had several limitations. First, the sample size was rather small, requiring further investigations as part of the CARE study. Second, the PTV is defined slightly different and dose fractionation schemes varied according to treatment group. Third, according to the clinical standard for CIRT at our institution, the α/β value was equal for the tumor and OAR, thereby underestimating radiobiological factors. Furthermore, the conversion of CIRT biological dose in EQD2 using the α/β used as input is an approximation not considering the local α and β values originating from the mixed radiation field of CIRT [40]. Nonetheless, the current study is the first to compare CIRT to VMAT as part of a randomized prospective trial, thereby increasing the body of evidence with relevant clinical data.

## Conclusions

Both VMAT and CIRT are feasible techniques for reirradiation of recurrent head and neck cancer. CIRT resulted in highly improved critical OAR sparing compared to VMAT across all head and neck cancer reirradiation scenarios despite an increased prescription dose. Pattern of local failure analysis revealed potential clinical pitfalls with regard to image-guidance and biological dose-optimization. Therefore, continued investigations within the CARE study will consider novel imaging and dose recalculation strategies for treatment planning.

## Supplementary Information

**Additional file 1: Tab. S3.** Target dose-volume comparison of reirradiation with CIRT vs. VMAT in recurrent head and neck cancer. Relative dose differences are specified in percent of 60 Gy equivalent dose in 2 Gy fractions. **Tab. S4.** Organs at risk dose-volume comparison of reirradiation with CIRT vs. VMAT in recurrent head and neck cancer. Relative dose differences are specified in percent of 60 Gy equivalent dose in 2 Gy fractions. **Tab. S5.** Clinical goals comparison of reirradiation with CIRT vs. VMAT in recurrent head and neck cancer. **Fig. S5.** 57-year-old male patient with recurrent nasopharyngeal cancer treated with 51 Gy (RBE) CIRT (D–F) around 0.7 years after prior radiotherapy with 74 Gy. CIRT yielded significant clinical benefits w.r.t. the spinal cord (− 29.5% Dmax) compared to VMAT (A–C). The patient developed type E local failure (> CTV + 5 mm), delineated on the planning CT (D–F), caused by aberrant areas of recurrence. **Fig. S6.** 72-year-old female patient with recurrent paranasal sinus cancer treated with 60 Gy VMAT (A–C) around 1 year after prior radiotherapy with 66 Gy. CIRT (D–F) yielded significant clinical benefits w.r.t. the brainstem (− 19.7% Dmax), ipsilateral eye (− 27.0% Dmean) and ipsilateral inner ear (− 13.3% Dmean). The patient developed type B and E (> CTV + 5 mm) local failure, delineated on the planning CT (A–C), caused by overgrown recurrence and aberrant areas of recurrence. **Fig. S7.** 54-year-old male patient with recurrent nasopharyngeal cancer treated with 60 Gy VMAT (A–C) around 4.5 years after prior radiotherapy with 64 Gy. CIRT (D–F) yielded significant clinical benefits w.r.t. the brainstem (− 37.0% Dmax), ipsilateral inner ear (− 26.8% Dmean) and contralateral inner ear (− 20.2% Dmean). The patient developed no local failure during follow-up. **Fig. S8.** 54-year-old male patient with recurrent paranasal sinus cancer treated with 54 Gy (RBE) CIRT (D–F) around 1.6 years after prior radiotherapy with 60 Gy. CIRT yielded significant clinical benefits w.r.t. the brainstem (− 43.5% Dmax) and the contralateral eye (− 17.7% Dmax) compared to VMAT (A–C). The patient developed type E local failure (< CTV + 5 mm), delineated on the planning CT (D–F), caused by improper risk assessment. **Fig. S9.** 52-year-old female patient with skull base recurrence treated with 60 Gy VMAT (A–C) around 1 year after prior radiotherapy with 70 Gy. CIRT (D–F) yielded significant clinical benefits w.r.t. the ipsilateral inner ear (− 13.8% Dmean) and the optic chiasma (− 13.5% Dmax) but not the brainstem (+ 2.2% Dmax). The patient developed type E local failure (> CTV + 5 mm), delineated on the planning CT (A–C), caused by aberrant areas of recurrence. **Fig. S10.** 49-year-old male patient with recurrent nasopharyngeal cancer treated with 60 Gy VMAT (A–C) around 3.3 years after prior radiotherapy with 64 Gy. CIRT (D–F) yielded significant clinical benefits w.r.t. the optic chiasma (− 50.7% Dmax), ipsilateral optic nerve (− 29.3% Dmax) and ipsilateral inner ear (− 28.0% Dmean). The patient developed type B local failure, delineated on the planning CT (A–C), caused by dosimetric failure due to direct contact of the tumor to the brainstem. CIRT was non-superior with regard to gross tumor volume coverage next to the brainstem. **Fig. S11.** 61-year-old male patient with hypopharyngeal recurrence treated with 51 Gy (RBE) CIRT (D–F) around 3.6 years after previous radiotherapy with 60 Gy. CIRT yielded significant clinical benefits w.r.t. the spinal cord (− 23.8% Dmax) compared to VMAT (A–C). The patient developed type A local failure in the central high-dose region of the CTV, caused by biological and/or dosimetric failure. The recurrent tumor (rGTV) and its centroid were delineated in black/red and mapped to the planning CT (D–F). Dose recalculation with the modified microdosimetric model showed no relevant changes compared to the local effect model I. **Fig. S12.** 50-year-old male patient with recurrent nasal cavity cancer treated with 51 Gy (RBE) CIRT (D–F) around 1.2 years after prior radiotherapy with 66 Gy. CIRT yielded significant clinical benefits w.r.t. the optic chiasma (− 25.0% Dmax), ipsilateral/contralateral optic nerve (− 41.0% Dmax) and ipsilateral eye (− 40.2% Dmean) compared to VMAT (A–C). The patient developed type B and E local failure, delineated on the planning CT (D–F), caused overgrown recurrence and aberrant areas of recurrence. **Fig. S13.** 69-year-old male patient with recurrent oropharyngeal cancer treated with 54 Gy (RBE) CIRT (D–F) around 17.9 years after prior radiotherapy with 66 Gy. CIRT yielded significant clinical benefits w.r.t. the spinal cord (− 25.8% Dmax) but not the mandible (+ 7.8% Dmax; − 10.2% Dmean) compared to VMAT (A–C). The patient developed no local failure during follow-up.

## Data Availability

The data that support the findings of this study are available from the corresponding author on reasonable request.

## Abbreviations

AC: Adenocarcinoma
CIRT: Carbon ion radiotherapy
CT: Computed tomography
CTV: Clinical target volume
EQD2: Equivalent dose in 2 Gy fractions
GTV: Gross tumor volume
KPS: Karnofsky Performance Score
LEM: Local effect model
LET: Linear energy transfer
MEC: Mucoepidermoid carcinoma
mMKM: Modified microdosimetric kinetic model
MRI: Magnetic resonance imaging
NTCP: Normal tissue complication probability
OAR: Organ at risk
PET: Positron emission tomography
PTV: Planning target volume
RBE: Relative biological effectiveness
rGTV: Recurrent macroscopic tumor
ReRT: Reirradiation
RT: Radiotherapy
SCC: Squamous cell carcinoma
VMAT: Volume modulated arc therapy
