# Supplementary figures and images for: Ways to unravel the clinical potential of carbon ions for head and neck cancer reirradiation: dosimetric comparison and local failure pattern analysis as part of the prospective randomized CARE trial
Source: Radiat Oncol. 2022 Jul 8;17:121. doi: 10.1186/s13014-022-02093-4 (PMC9264522; doi:10.1186/s13014-022-02093-4)

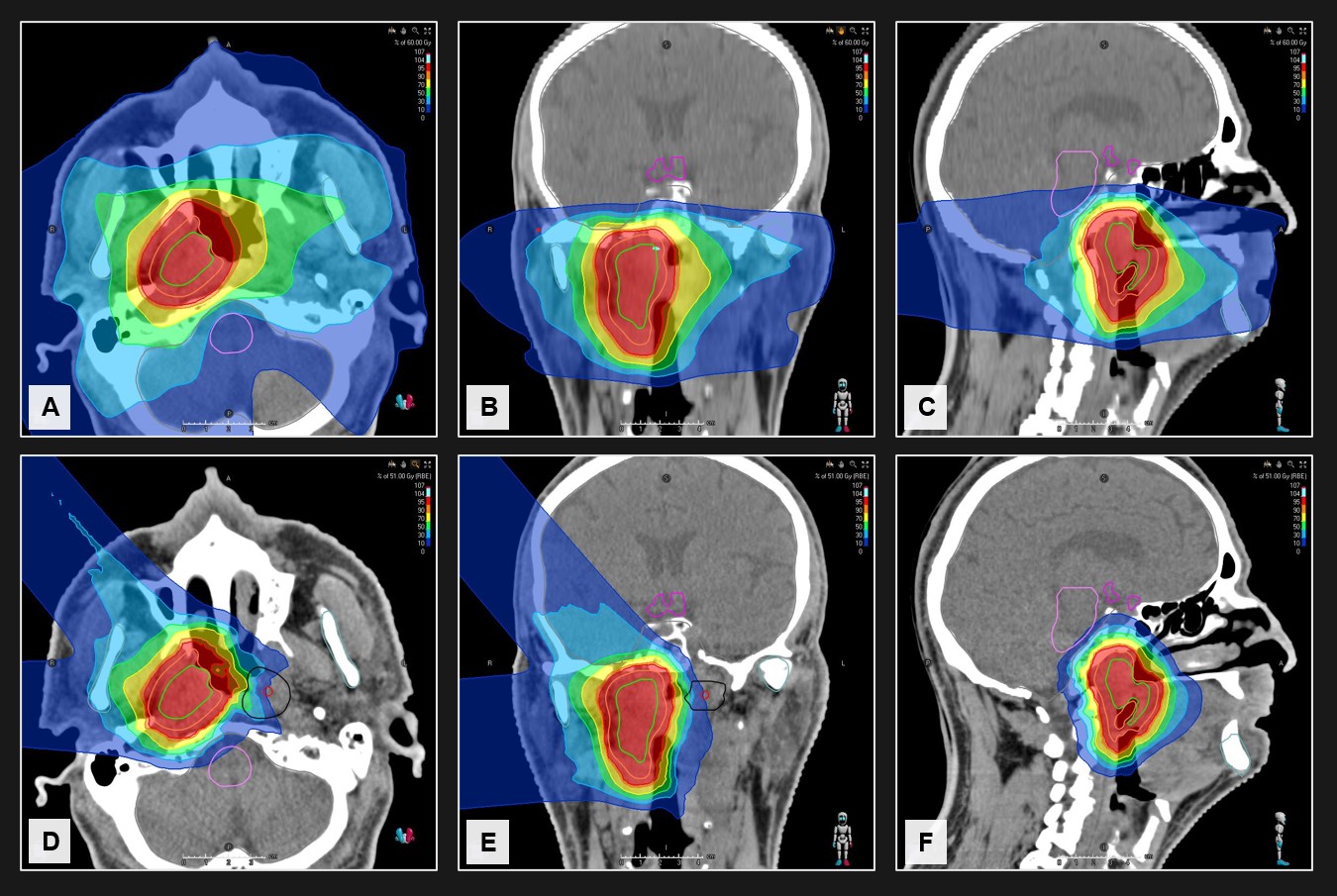

Supplement: Supplementary file 1 — Additional file 1: Tab. S3. Target dose-volume comparison of reirradiation with CIRT vs. VMAT in recurrent head and neck cancer. Relative dose differences are specified in percent of 60 Gy equivalent dose in 2 Gy fractions. Tab. S4. Organs at risk dose-volume comparison of reirradiation with CIRT vs. VMAT in recurrent head and neck cancer. Relative dose differences are specified in percent of 60 Gy equivalent dose in 2 Gy fractions. Tab. S5. Clinical goals comparison of reirradiation with CIRT vs. VMAT in recurrent head and neck cancer. Fig. S5. 57-year-old male patient with recurrent nasopharyngeal cancer treated with 51 Gy (RBE) CIRT (D–F) around 0.7 years after prior radiotherapy with 74 Gy. CIRT yielded significant clinical benefits w.r.t. the spinal cord (− 29.5% Dmax) compared to VMAT (A–C). The patient developed type E local failure (> CTV + 5 mm), delineated on the planning CT (D–F), caused by aberrant areas of recurrence. Fig. S6. 72-year-old female patient with recurrent paranasal sinus cancer treated with 60 Gy VMAT (A–C) around 1 year after prior radiotherapy with 66 Gy. CIRT (D–F) yielded significant clinical benefits w.r.t. the brainstem (− 19.7% Dmax), ipsilateral eye (− 27.0% Dmean) and ipsilateral inner ear (− 13.3% Dmean). The patient developed type B and E (> CTV + 5 mm) local failure, delineated on the planning CT (A–C), caused by overgrown recurrence and aberrant areas of recurrence. Fig. S7. 54-year-old male patient with recurrent nasopharyngeal cancer treated with 60 Gy VMAT (A–C) around 4.5 years after prior radiotherapy with 64 Gy. CIRT (D–F) yielded significant clinical benefits w.r.t. the brainstem (− 37.0% Dmax), ipsilateral inner ear (− 26.8% Dmean) and contralateral inner ear (− 20.2% Dmean). The patient developed no local failure during follow-up. Fig. S8. 54-year-old male patient with recurrent paranasal sinus cancer treated with 54 Gy (RBE) CIRT (D–F) around 1.6 years after prior radiotherapy with 60 Gy. CIRT yielded significant clin [file 13014_2022_2093_MOESM1_ESM.zip › Figure_05.jpg]

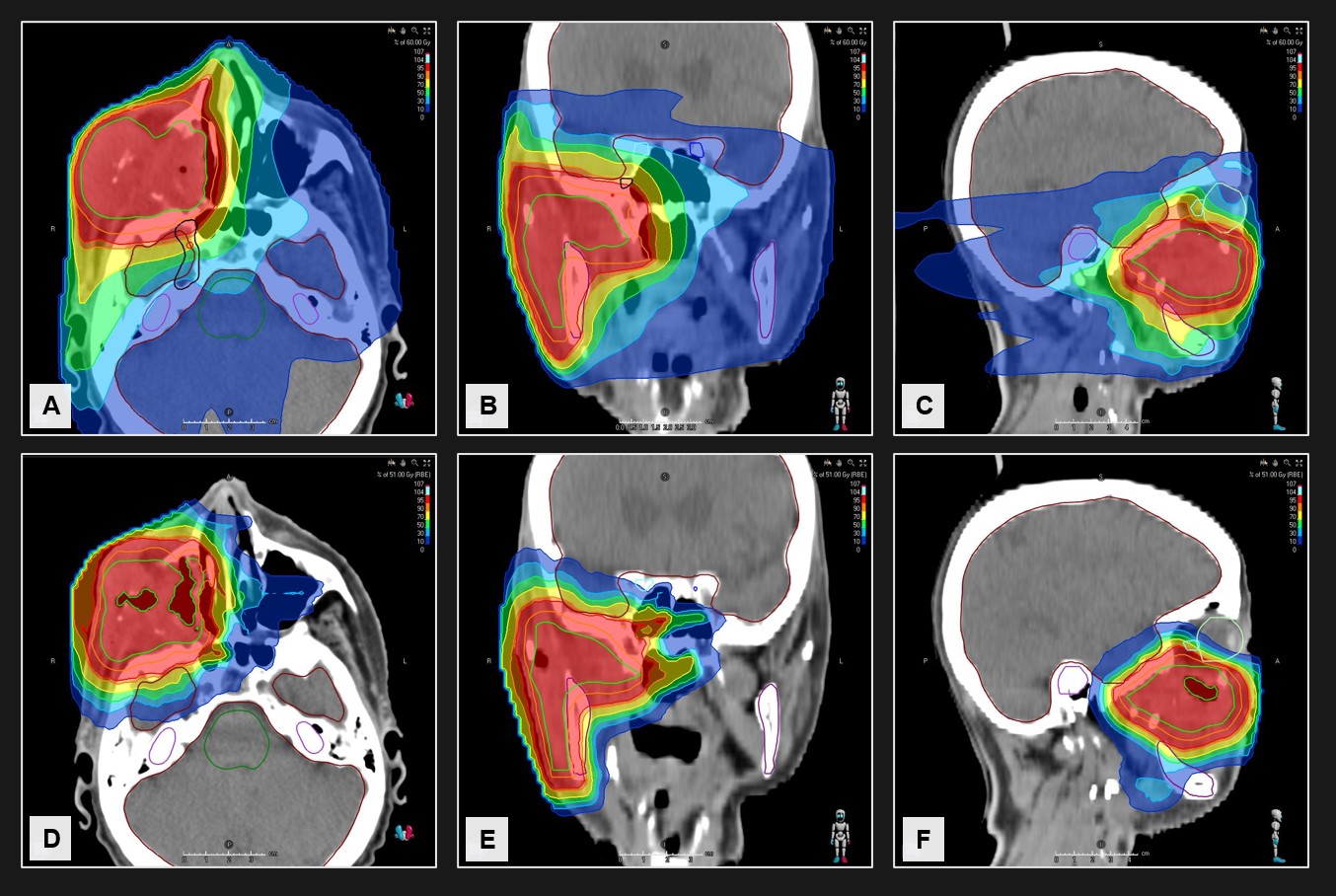

Supplement: Supplementary file 1 — Additional file 1: Tab. S3. Target dose-volume comparison of reirradiation with CIRT vs. VMAT in recurrent head and neck cancer. Relative dose differences are specified in percent of 60 Gy equivalent dose in 2 Gy fractions. Tab. S4. Organs at risk dose-volume comparison of reirradiation with CIRT vs. VMAT in recurrent head and neck cancer. Relative dose differences are specified in percent of 60 Gy equivalent dose in 2 Gy fractions. Tab. S5. Clinical goals comparison of reirradiation with CIRT vs. VMAT in recurrent head and neck cancer. Fig. S5. 57-year-old male patient with recurrent nasopharyngeal cancer treated with 51 Gy (RBE) CIRT (D–F) around 0.7 years after prior radiotherapy with 74 Gy. CIRT yielded significant clinical benefits w.r.t. the spinal cord (− 29.5% Dmax) compared to VMAT (A–C). The patient developed type E local failure (> CTV + 5 mm), delineated on the planning CT (D–F), caused by aberrant areas of recurrence. Fig. S6. 72-year-old female patient with recurrent paranasal sinus cancer treated with 60 Gy VMAT (A–C) around 1 year after prior radiotherapy with 66 Gy. CIRT (D–F) yielded significant clinical benefits w.r.t. the brainstem (− 19.7% Dmax), ipsilateral eye (− 27.0% Dmean) and ipsilateral inner ear (− 13.3% Dmean). The patient developed type B and E (> CTV + 5 mm) local failure, delineated on the planning CT (A–C), caused by overgrown recurrence and aberrant areas of recurrence. Fig. S7. 54-year-old male patient with recurrent nasopharyngeal cancer treated with 60 Gy VMAT (A–C) around 4.5 years after prior radiotherapy with 64 Gy. CIRT (D–F) yielded significant clinical benefits w.r.t. the brainstem (− 37.0% Dmax), ipsilateral inner ear (− 26.8% Dmean) and contralateral inner ear (− 20.2% Dmean). The patient developed no local failure during follow-up. Fig. S8. 54-year-old male patient with recurrent paranasal sinus cancer treated with 54 Gy (RBE) CIRT (D–F) around 1.6 years after prior radiotherapy with 60 Gy. CIRT yielded significant clin [file 13014_2022_2093_MOESM1_ESM.zip › Figure_06.jpg]

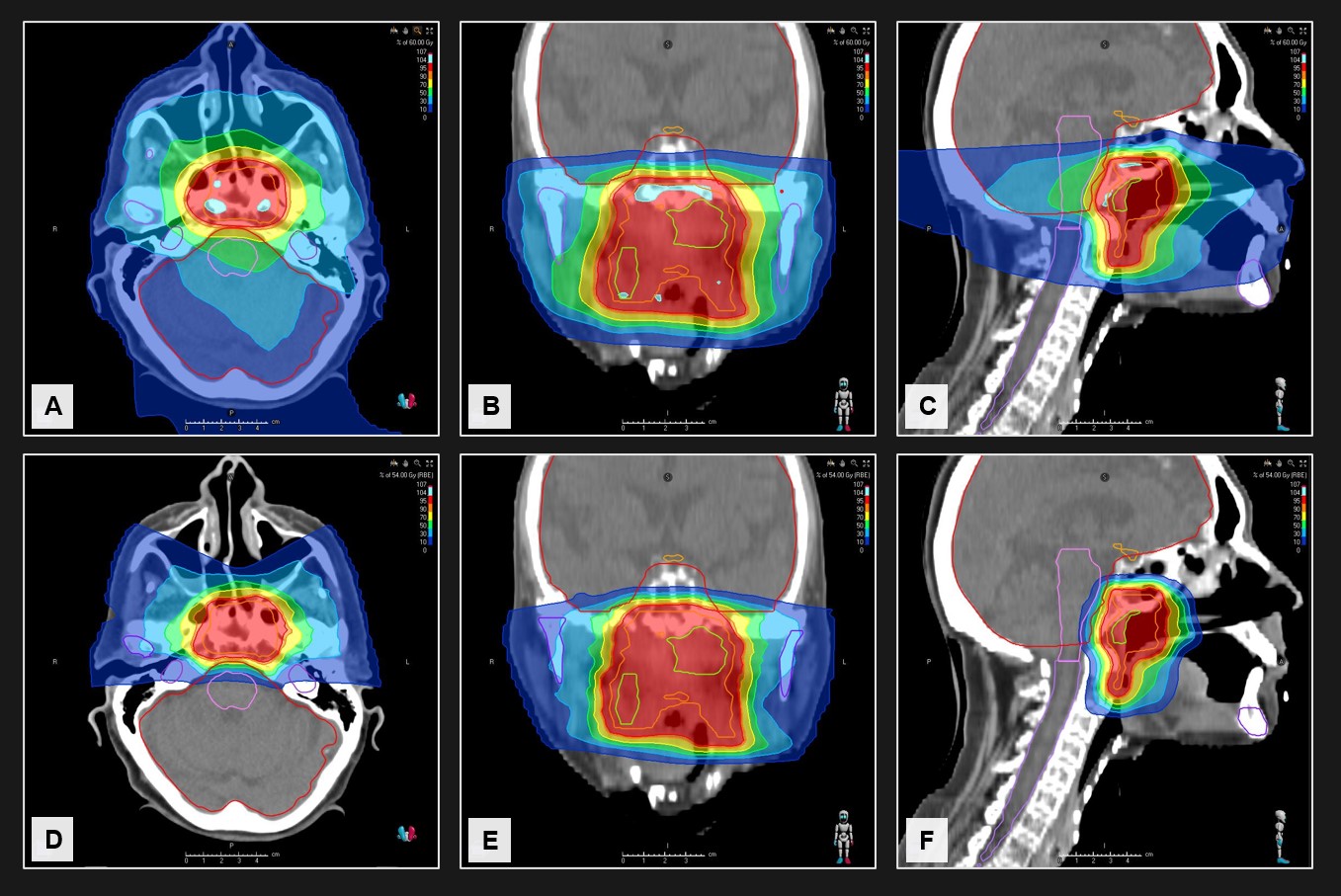

Supplement: Supplementary file 1 — Additional file 1: Tab. S3. Target dose-volume comparison of reirradiation with CIRT vs. VMAT in recurrent head and neck cancer. Relative dose differences are specified in percent of 60 Gy equivalent dose in 2 Gy fractions. Tab. S4. Organs at risk dose-volume comparison of reirradiation with CIRT vs. VMAT in recurrent head and neck cancer. Relative dose differences are specified in percent of 60 Gy equivalent dose in 2 Gy fractions. Tab. S5. Clinical goals comparison of reirradiation with CIRT vs. VMAT in recurrent head and neck cancer. Fig. S5. 57-year-old male patient with recurrent nasopharyngeal cancer treated with 51 Gy (RBE) CIRT (D–F) around 0.7 years after prior radiotherapy with 74 Gy. CIRT yielded significant clinical benefits w.r.t. the spinal cord (− 29.5% Dmax) compared to VMAT (A–C). The patient developed type E local failure (> CTV + 5 mm), delineated on the planning CT (D–F), caused by aberrant areas of recurrence. Fig. S6. 72-year-old female patient with recurrent paranasal sinus cancer treated with 60 Gy VMAT (A–C) around 1 year after prior radiotherapy with 66 Gy. CIRT (D–F) yielded significant clinical benefits w.r.t. the brainstem (− 19.7% Dmax), ipsilateral eye (− 27.0% Dmean) and ipsilateral inner ear (− 13.3% Dmean). The patient developed type B and E (> CTV + 5 mm) local failure, delineated on the planning CT (A–C), caused by overgrown recurrence and aberrant areas of recurrence. Fig. S7. 54-year-old male patient with recurrent nasopharyngeal cancer treated with 60 Gy VMAT (A–C) around 4.5 years after prior radiotherapy with 64 Gy. CIRT (D–F) yielded significant clinical benefits w.r.t. the brainstem (− 37.0% Dmax), ipsilateral inner ear (− 26.8% Dmean) and contralateral inner ear (− 20.2% Dmean). The patient developed no local failure during follow-up. Fig. S8. 54-year-old male patient with recurrent paranasal sinus cancer treated with 54 Gy (RBE) CIRT (D–F) around 1.6 years after prior radiotherapy with 60 Gy. CIRT yielded significant clin [file 13014_2022_2093_MOESM1_ESM.zip › Figure_07.jpg]

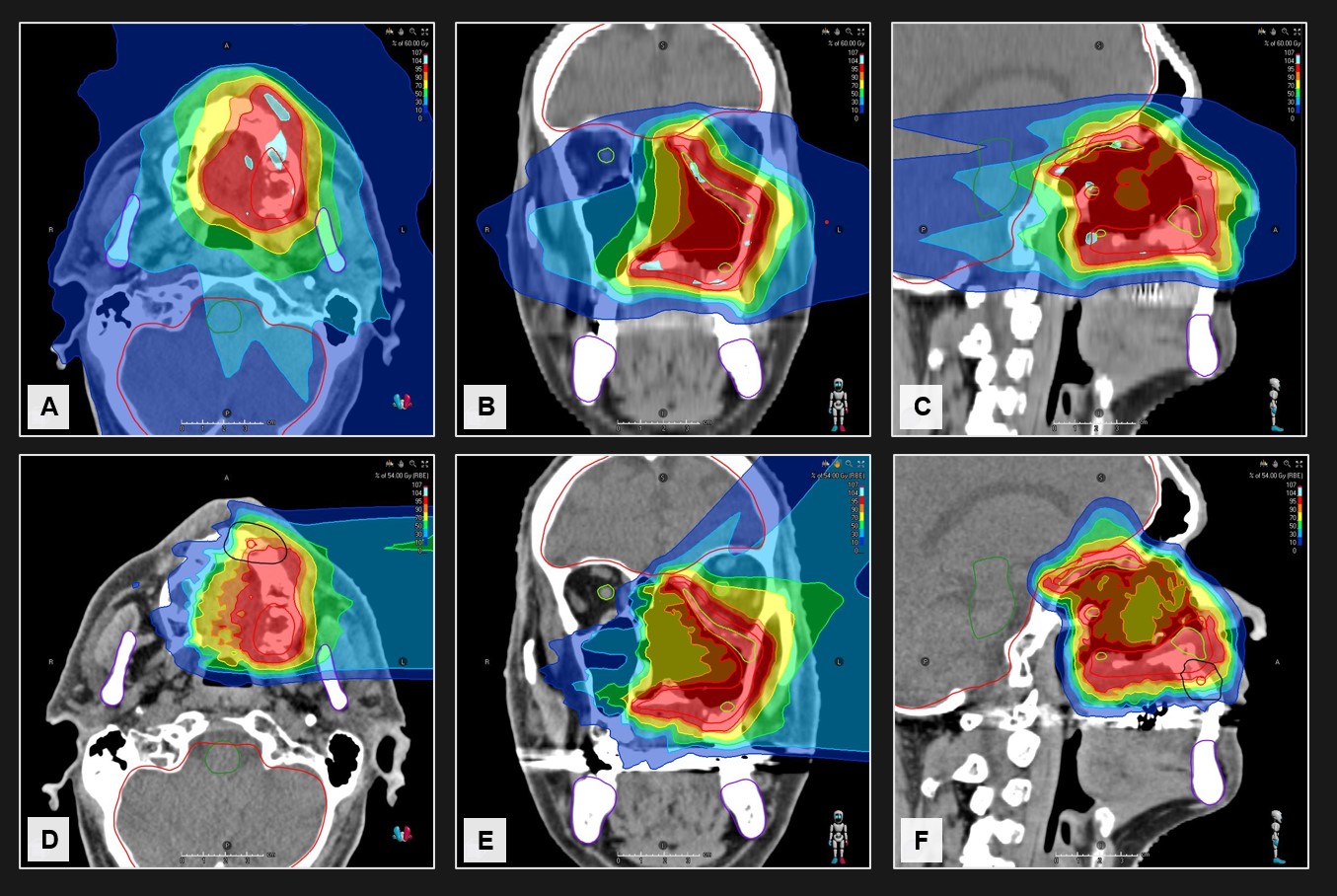

Supplement: Supplementary file 1 — Additional file 1: Tab. S3. Target dose-volume comparison of reirradiation with CIRT vs. VMAT in recurrent head and neck cancer. Relative dose differences are specified in percent of 60 Gy equivalent dose in 2 Gy fractions. Tab. S4. Organs at risk dose-volume comparison of reirradiation with CIRT vs. VMAT in recurrent head and neck cancer. Relative dose differences are specified in percent of 60 Gy equivalent dose in 2 Gy fractions. Tab. S5. Clinical goals comparison of reirradiation with CIRT vs. VMAT in recurrent head and neck cancer. Fig. S5. 57-year-old male patient with recurrent nasopharyngeal cancer treated with 51 Gy (RBE) CIRT (D–F) around 0.7 years after prior radiotherapy with 74 Gy. CIRT yielded significant clinical benefits w.r.t. the spinal cord (− 29.5% Dmax) compared to VMAT (A–C). The patient developed type E local failure (> CTV + 5 mm), delineated on the planning CT (D–F), caused by aberrant areas of recurrence. Fig. S6. 72-year-old female patient with recurrent paranasal sinus cancer treated with 60 Gy VMAT (A–C) around 1 year after prior radiotherapy with 66 Gy. CIRT (D–F) yielded significant clinical benefits w.r.t. the brainstem (− 19.7% Dmax), ipsilateral eye (− 27.0% Dmean) and ipsilateral inner ear (− 13.3% Dmean). The patient developed type B and E (> CTV + 5 mm) local failure, delineated on the planning CT (A–C), caused by overgrown recurrence and aberrant areas of recurrence. Fig. S7. 54-year-old male patient with recurrent nasopharyngeal cancer treated with 60 Gy VMAT (A–C) around 4.5 years after prior radiotherapy with 64 Gy. CIRT (D–F) yielded significant clinical benefits w.r.t. the brainstem (− 37.0% Dmax), ipsilateral inner ear (− 26.8% Dmean) and contralateral inner ear (− 20.2% Dmean). The patient developed no local failure during follow-up. Fig. S8. 54-year-old male patient with recurrent paranasal sinus cancer treated with 54 Gy (RBE) CIRT (D–F) around 1.6 years after prior radiotherapy with 60 Gy. CIRT yielded significant clin [file 13014_2022_2093_MOESM1_ESM.zip › Figure_08.jpg]

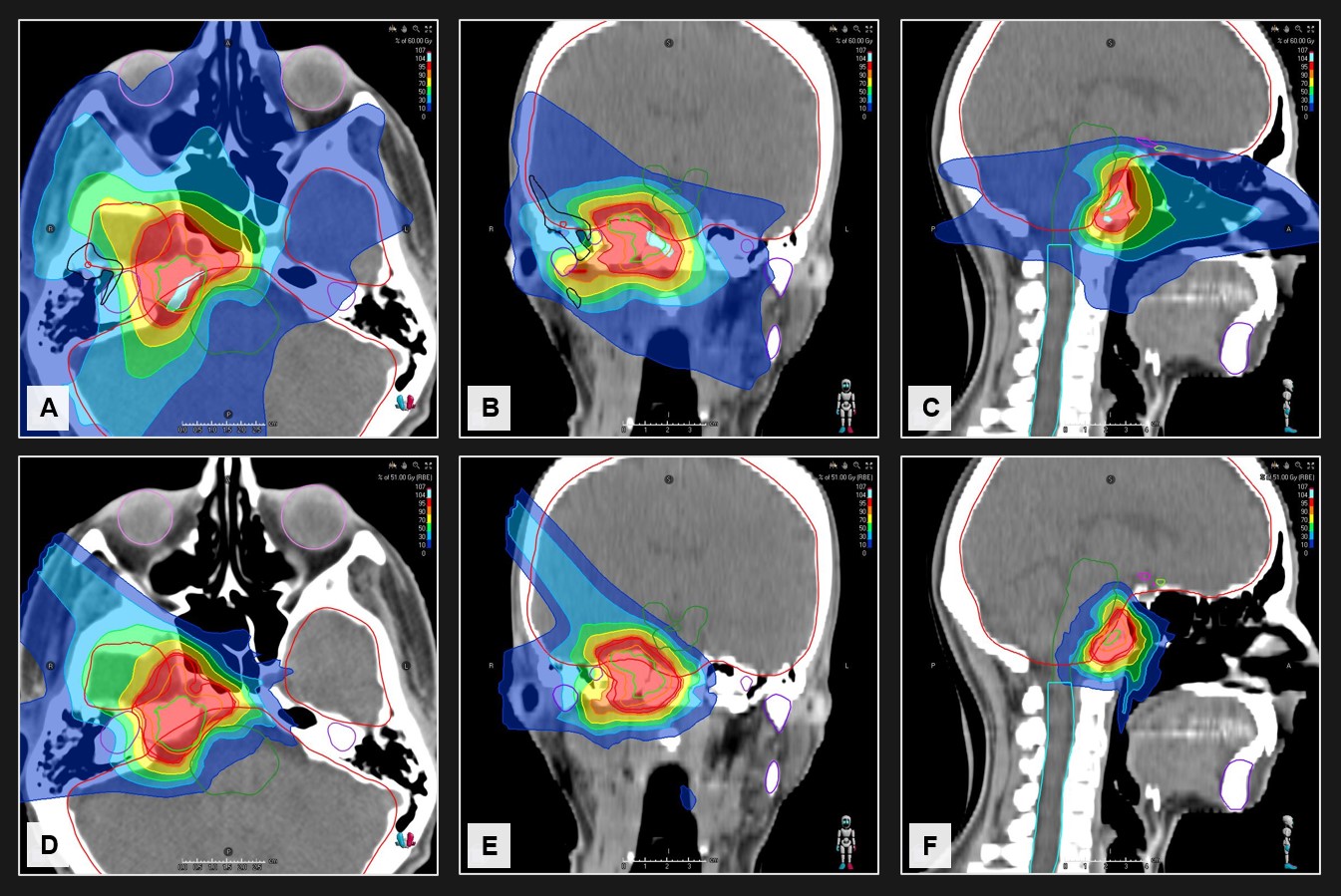

Supplement: Supplementary file 1 — Additional file 1: Tab. S3. Target dose-volume comparison of reirradiation with CIRT vs. VMAT in recurrent head and neck cancer. Relative dose differences are specified in percent of 60 Gy equivalent dose in 2 Gy fractions. Tab. S4. Organs at risk dose-volume comparison of reirradiation with CIRT vs. VMAT in recurrent head and neck cancer. Relative dose differences are specified in percent of 60 Gy equivalent dose in 2 Gy fractions. Tab. S5. Clinical goals comparison of reirradiation with CIRT vs. VMAT in recurrent head and neck cancer. Fig. S5. 57-year-old male patient with recurrent nasopharyngeal cancer treated with 51 Gy (RBE) CIRT (D–F) around 0.7 years after prior radiotherapy with 74 Gy. CIRT yielded significant clinical benefits w.r.t. the spinal cord (− 29.5% Dmax) compared to VMAT (A–C). The patient developed type E local failure (> CTV + 5 mm), delineated on the planning CT (D–F), caused by aberrant areas of recurrence. Fig. S6. 72-year-old female patient with recurrent paranasal sinus cancer treated with 60 Gy VMAT (A–C) around 1 year after prior radiotherapy with 66 Gy. CIRT (D–F) yielded significant clinical benefits w.r.t. the brainstem (− 19.7% Dmax), ipsilateral eye (− 27.0% Dmean) and ipsilateral inner ear (− 13.3% Dmean). The patient developed type B and E (> CTV + 5 mm) local failure, delineated on the planning CT (A–C), caused by overgrown recurrence and aberrant areas of recurrence. Fig. S7. 54-year-old male patient with recurrent nasopharyngeal cancer treated with 60 Gy VMAT (A–C) around 4.5 years after prior radiotherapy with 64 Gy. CIRT (D–F) yielded significant clinical benefits w.r.t. the brainstem (− 37.0% Dmax), ipsilateral inner ear (− 26.8% Dmean) and contralateral inner ear (− 20.2% Dmean). The patient developed no local failure during follow-up. Fig. S8. 54-year-old male patient with recurrent paranasal sinus cancer treated with 54 Gy (RBE) CIRT (D–F) around 1.6 years after prior radiotherapy with 60 Gy. CIRT yielded significant clin [file 13014_2022_2093_MOESM1_ESM.zip › Figure_09.jpg]

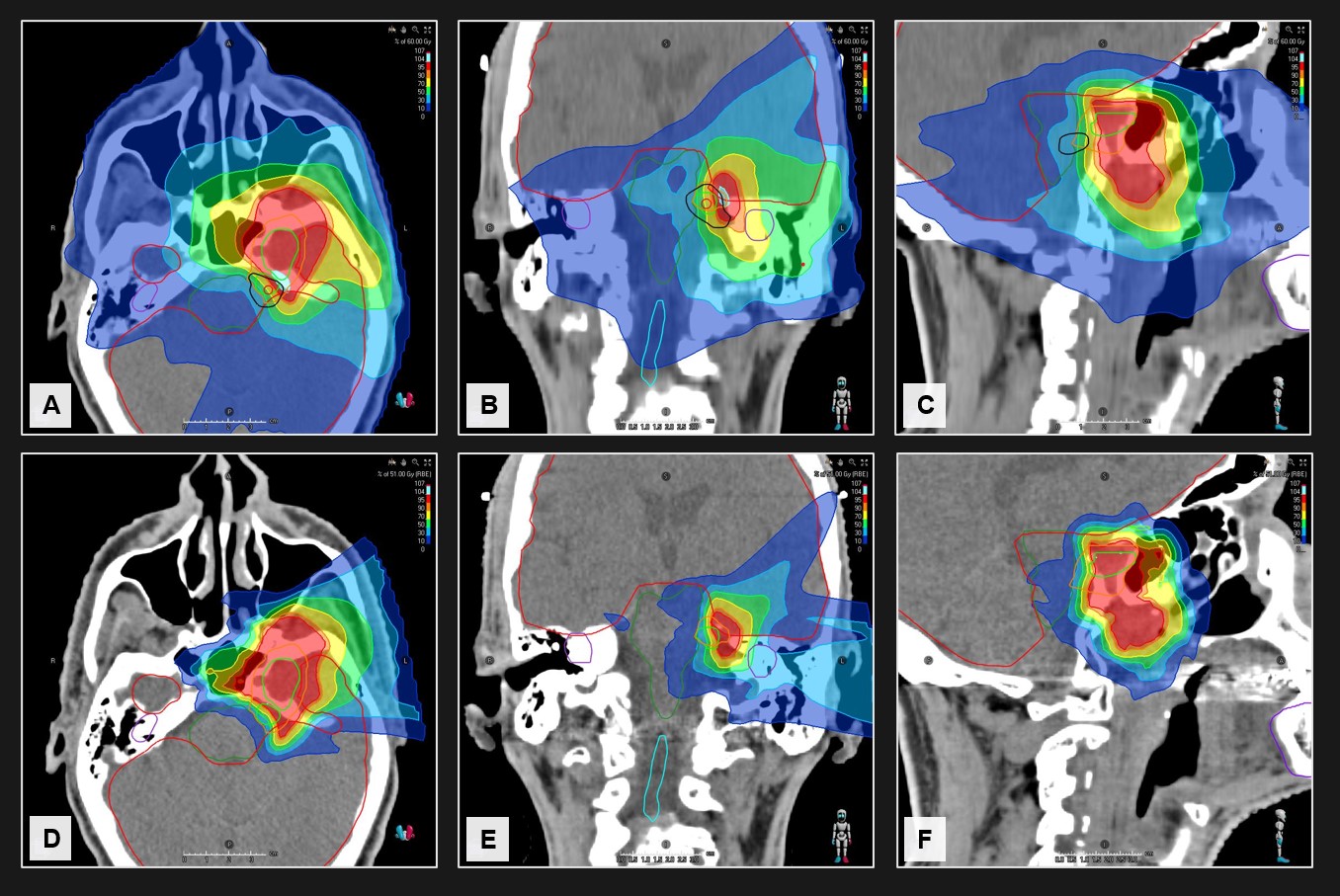

Supplement: Supplementary file 1 — Additional file 1: Tab. S3. Target dose-volume comparison of reirradiation with CIRT vs. VMAT in recurrent head and neck cancer. Relative dose differences are specified in percent of 60 Gy equivalent dose in 2 Gy fractions. Tab. S4. Organs at risk dose-volume comparison of reirradiation with CIRT vs. VMAT in recurrent head and neck cancer. Relative dose differences are specified in percent of 60 Gy equivalent dose in 2 Gy fractions. Tab. S5. Clinical goals comparison of reirradiation with CIRT vs. VMAT in recurrent head and neck cancer. Fig. S5. 57-year-old male patient with recurrent nasopharyngeal cancer treated with 51 Gy (RBE) CIRT (D–F) around 0.7 years after prior radiotherapy with 74 Gy. CIRT yielded significant clinical benefits w.r.t. the spinal cord (− 29.5% Dmax) compared to VMAT (A–C). The patient developed type E local failure (> CTV + 5 mm), delineated on the planning CT (D–F), caused by aberrant areas of recurrence. Fig. S6. 72-year-old female patient with recurrent paranasal sinus cancer treated with 60 Gy VMAT (A–C) around 1 year after prior radiotherapy with 66 Gy. CIRT (D–F) yielded significant clinical benefits w.r.t. the brainstem (− 19.7% Dmax), ipsilateral eye (− 27.0% Dmean) and ipsilateral inner ear (− 13.3% Dmean). The patient developed type B and E (> CTV + 5 mm) local failure, delineated on the planning CT (A–C), caused by overgrown recurrence and aberrant areas of recurrence. Fig. S7. 54-year-old male patient with recurrent nasopharyngeal cancer treated with 60 Gy VMAT (A–C) around 4.5 years after prior radiotherapy with 64 Gy. CIRT (D–F) yielded significant clinical benefits w.r.t. the brainstem (− 37.0% Dmax), ipsilateral inner ear (− 26.8% Dmean) and contralateral inner ear (− 20.2% Dmean). The patient developed no local failure during follow-up. Fig. S8. 54-year-old male patient with recurrent paranasal sinus cancer treated with 54 Gy (RBE) CIRT (D–F) around 1.6 years after prior radiotherapy with 60 Gy. CIRT yielded significant clin [file 13014_2022_2093_MOESM1_ESM.zip › Figure_10.jpg]

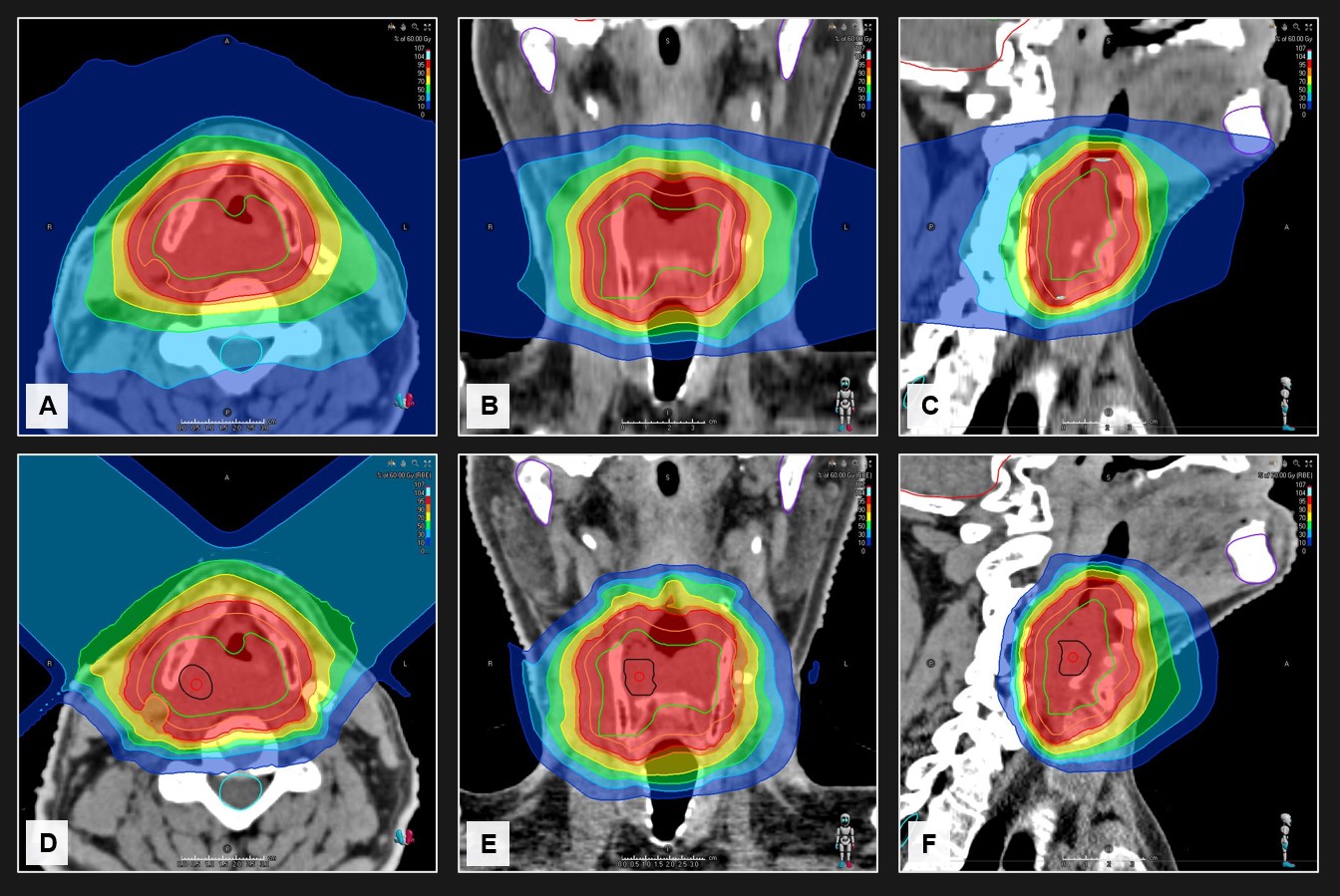

Supplement: Supplementary file 1 — Additional file 1: Tab. S3. Target dose-volume comparison of reirradiation with CIRT vs. VMAT in recurrent head and neck cancer. Relative dose differences are specified in percent of 60 Gy equivalent dose in 2 Gy fractions. Tab. S4. Organs at risk dose-volume comparison of reirradiation with CIRT vs. VMAT in recurrent head and neck cancer. Relative dose differences are specified in percent of 60 Gy equivalent dose in 2 Gy fractions. Tab. S5. Clinical goals comparison of reirradiation with CIRT vs. VMAT in recurrent head and neck cancer. Fig. S5. 57-year-old male patient with recurrent nasopharyngeal cancer treated with 51 Gy (RBE) CIRT (D–F) around 0.7 years after prior radiotherapy with 74 Gy. CIRT yielded significant clinical benefits w.r.t. the spinal cord (− 29.5% Dmax) compared to VMAT (A–C). The patient developed type E local failure (> CTV + 5 mm), delineated on the planning CT (D–F), caused by aberrant areas of recurrence. Fig. S6. 72-year-old female patient with recurrent paranasal sinus cancer treated with 60 Gy VMAT (A–C) around 1 year after prior radiotherapy with 66 Gy. CIRT (D–F) yielded significant clinical benefits w.r.t. the brainstem (− 19.7% Dmax), ipsilateral eye (− 27.0% Dmean) and ipsilateral inner ear (− 13.3% Dmean). The patient developed type B and E (> CTV + 5 mm) local failure, delineated on the planning CT (A–C), caused by overgrown recurrence and aberrant areas of recurrence. Fig. S7. 54-year-old male patient with recurrent nasopharyngeal cancer treated with 60 Gy VMAT (A–C) around 4.5 years after prior radiotherapy with 64 Gy. CIRT (D–F) yielded significant clinical benefits w.r.t. the brainstem (− 37.0% Dmax), ipsilateral inner ear (− 26.8% Dmean) and contralateral inner ear (− 20.2% Dmean). The patient developed no local failure during follow-up. Fig. S8. 54-year-old male patient with recurrent paranasal sinus cancer treated with 54 Gy (RBE) CIRT (D–F) around 1.6 years after prior radiotherapy with 60 Gy. CIRT yielded significant clin [file 13014_2022_2093_MOESM1_ESM.zip › Figure_11.jpg]

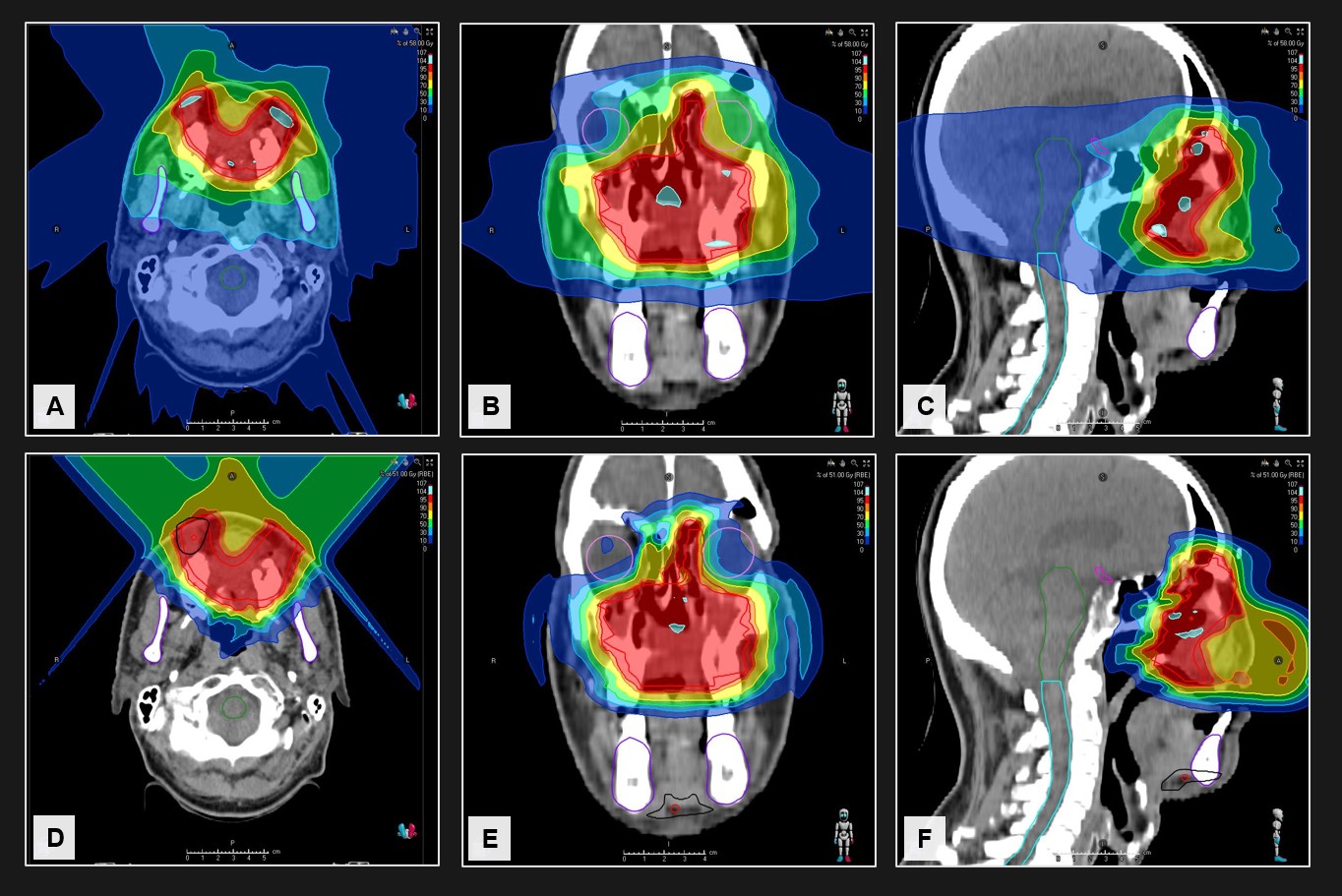

Supplement: Supplementary file 1 — Additional file 1: Tab. S3. Target dose-volume comparison of reirradiation with CIRT vs. VMAT in recurrent head and neck cancer. Relative dose differences are specified in percent of 60 Gy equivalent dose in 2 Gy fractions. Tab. S4. Organs at risk dose-volume comparison of reirradiation with CIRT vs. VMAT in recurrent head and neck cancer. Relative dose differences are specified in percent of 60 Gy equivalent dose in 2 Gy fractions. Tab. S5. Clinical goals comparison of reirradiation with CIRT vs. VMAT in recurrent head and neck cancer. Fig. S5. 57-year-old male patient with recurrent nasopharyngeal cancer treated with 51 Gy (RBE) CIRT (D–F) around 0.7 years after prior radiotherapy with 74 Gy. CIRT yielded significant clinical benefits w.r.t. the spinal cord (− 29.5% Dmax) compared to VMAT (A–C). The patient developed type E local failure (> CTV + 5 mm), delineated on the planning CT (D–F), caused by aberrant areas of recurrence. Fig. S6. 72-year-old female patient with recurrent paranasal sinus cancer treated with 60 Gy VMAT (A–C) around 1 year after prior radiotherapy with 66 Gy. CIRT (D–F) yielded significant clinical benefits w.r.t. the brainstem (− 19.7% Dmax), ipsilateral eye (− 27.0% Dmean) and ipsilateral inner ear (− 13.3% Dmean). The patient developed type B and E (> CTV + 5 mm) local failure, delineated on the planning CT (A–C), caused by overgrown recurrence and aberrant areas of recurrence. Fig. S7. 54-year-old male patient with recurrent nasopharyngeal cancer treated with 60 Gy VMAT (A–C) around 4.5 years after prior radiotherapy with 64 Gy. CIRT (D–F) yielded significant clinical benefits w.r.t. the brainstem (− 37.0% Dmax), ipsilateral inner ear (− 26.8% Dmean) and contralateral inner ear (− 20.2% Dmean). The patient developed no local failure during follow-up. Fig. S8. 54-year-old male patient with recurrent paranasal sinus cancer treated with 54 Gy (RBE) CIRT (D–F) around 1.6 years after prior radiotherapy with 60 Gy. CIRT yielded significant clin [file 13014_2022_2093_MOESM1_ESM.zip › Figure_12.jpg]

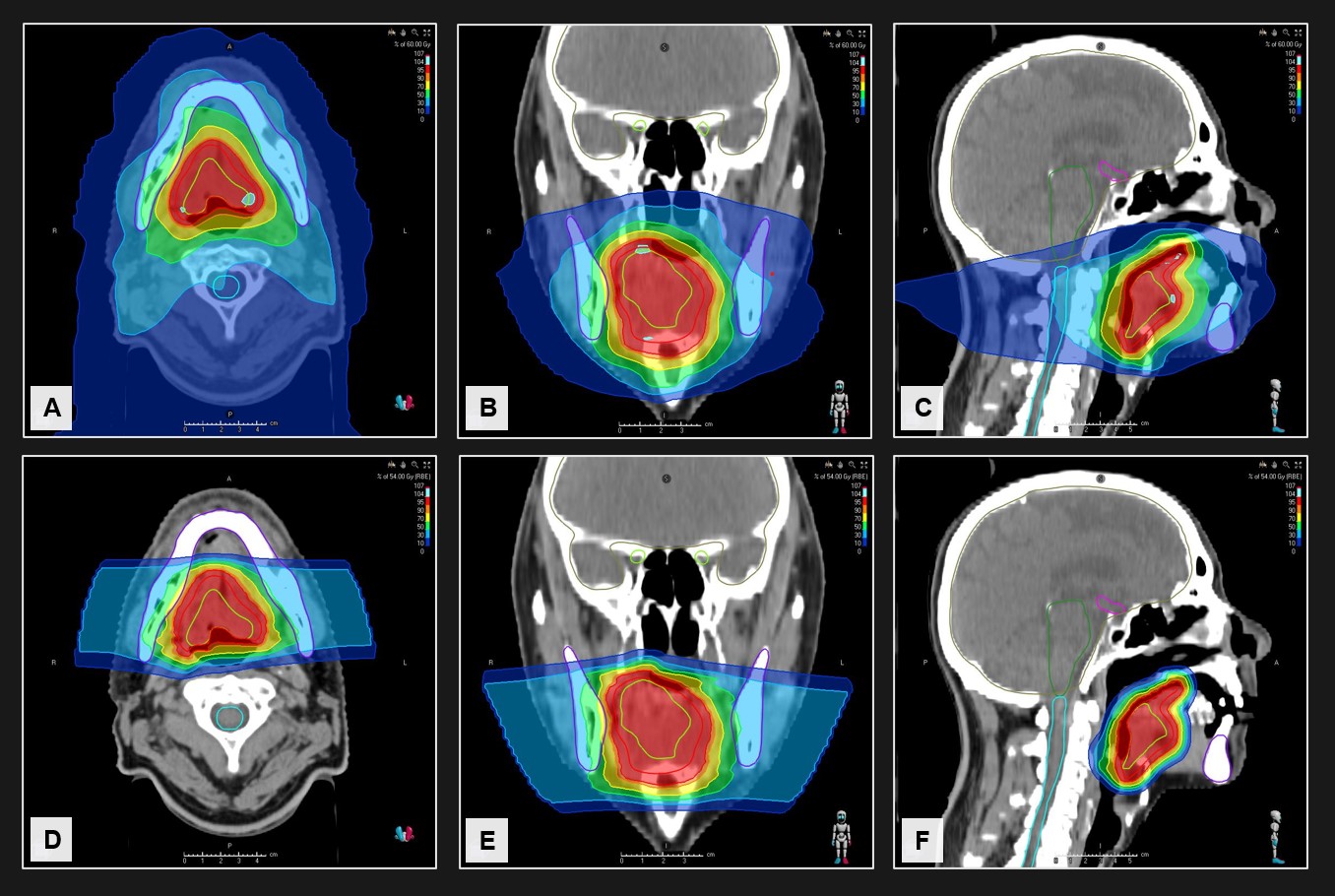

Supplement: Supplementary file 1 — Additional file 1: Tab. S3. Target dose-volume comparison of reirradiation with CIRT vs. VMAT in recurrent head and neck cancer. Relative dose differences are specified in percent of 60 Gy equivalent dose in 2 Gy fractions. Tab. S4. Organs at risk dose-volume comparison of reirradiation with CIRT vs. VMAT in recurrent head and neck cancer. Relative dose differences are specified in percent of 60 Gy equivalent dose in 2 Gy fractions. Tab. S5. Clinical goals comparison of reirradiation with CIRT vs. VMAT in recurrent head and neck cancer. Fig. S5. 57-year-old male patient with recurrent nasopharyngeal cancer treated with 51 Gy (RBE) CIRT (D–F) around 0.7 years after prior radiotherapy with 74 Gy. CIRT yielded significant clinical benefits w.r.t. the spinal cord (− 29.5% Dmax) compared to VMAT (A–C). The patient developed type E local failure (> CTV + 5 mm), delineated on the planning CT (D–F), caused by aberrant areas of recurrence. Fig. S6. 72-year-old female patient with recurrent paranasal sinus cancer treated with 60 Gy VMAT (A–C) around 1 year after prior radiotherapy with 66 Gy. CIRT (D–F) yielded significant clinical benefits w.r.t. the brainstem (− 19.7% Dmax), ipsilateral eye (− 27.0% Dmean) and ipsilateral inner ear (− 13.3% Dmean). The patient developed type B and E (> CTV + 5 mm) local failure, delineated on the planning CT (A–C), caused by overgrown recurrence and aberrant areas of recurrence. Fig. S7. 54-year-old male patient with recurrent nasopharyngeal cancer treated with 60 Gy VMAT (A–C) around 4.5 years after prior radiotherapy with 64 Gy. CIRT (D–F) yielded significant clinical benefits w.r.t. the brainstem (− 37.0% Dmax), ipsilateral inner ear (− 26.8% Dmean) and contralateral inner ear (− 20.2% Dmean). The patient developed no local failure during follow-up. Fig. S8. 54-year-old male patient with recurrent paranasal sinus cancer treated with 54 Gy (RBE) CIRT (D–F) around 1.6 years after prior radiotherapy with 60 Gy. CIRT yielded significant clin [file 13014_2022_2093_MOESM1_ESM.zip › Figure_13.jpg]
